# Synthesis of Graphene Oxide-Fe_3_O_4_ Based Nanocomposites Using the Mechanochemical Method and In Vitro Magnetic Hyperthermia

**DOI:** 10.3390/ijms20133368

**Published:** 2019-07-09

**Authors:** Venkatesha Narayanaswamy, Ihab M. Obaidat, Aleksandr S. Kamzin, Sachin Latiyan, Shilpee Jain, Hemant Kumar, Chandan Srivastava, Sulaiman Alaabed, Bashar Issa

**Affiliations:** 1 Department of Physics, United Arab Emirates University, Al-Ain 15551, United Arab Emirates; 2 Ioffe Physical Technical Institute, St. Petersburg 194021, Russia; 3 Center for Biosystems Science and Engineering, Indian Institute of Science, Bangalore 560012, India; 4 Materials Engineering, Indian Institute of Science, Bangalore 560012, India; 5 Department of Geology, United Arab Emirates University, Al-Ain 15551, United Arab Emirates; 6 Department of Medical Diagnostic Imaging, College of Health Sciences, University of Sharjah, Sharjah P.O. Box 27272, United Arab Emirates

**Keywords:** mechanochemical method, graphene oxide, hyperthermia

## Abstract

The study presented in this work consists of two parts: The first part is the synthesis of Graphene oxide-Fe_3_O_4_ nanocomposites by a mechanochemical method which, is a mechanical process that is likely to yield extremely heterogeneous particles. The second part includes a study on the efficacy of these Graphene oxide-Fe_3_O_4_ nanocomposites to kill cancerous cells. Iron powder, ball milled along with graphene oxide in a toluene medium, underwent a controlled oxidation process. Different phases of GO-Fe_3_O_4_ nanocomposites were obtained based on the composition used for milling. As synthesized nanocomposites were characterized by x-ray diffraction (XRD), alternating magnetic field (AFM), Raman spectroscopy, and a vibrating sample magnetometer (VSM). Additionally, the magnetic properties required to obtain high SAR values (Specific Absorption Rate-Power absorbed per unit mass of the magnetic nanocomposite in the presence of an applied magnetic field) for the composite were optimized by varying the milling time. Nanocomposites milled for different extents of time have shown differential behavior for magneto thermic heating. The magnetic composites synthesized by the ball milled method were able to retain the functional groups of graphene oxide. The efficacy of the magnetic nanocomposites for killing of cancerous cells is studied in vitro using HeLa cells in the presence of an AC (Alternating Current) magnetic field. The morphology of the HeLa cells subjected to 10 min of AC magnetic field changed considerably, indicating the death of the cells.

## 1. Introduction

Graphene oxide-Fe_3_O_4_ nanoparticle-based nanocomposites are widely investigated for various biomedical applications, like drug delivery, magnetic hyperthermia, and MRI (Magnetic Resonance Imaging) contrast agents [1,2,3,4,5]. Unique chemical and physical properties of graphene oxide-based nanocomposites enables us to design and engineer their structure as per requirements [6]. The presence of functional groups, i.e., –OH, –COOH, and –CHO, renders the easy attachment and release of various biomolecules and drugs [7]. Anticancer drugs can be delivered using graphene oxide-based composites as the carrier vehicle in response to change of pH or local heating via hyperthermia [8]. There are various studies where Graphene oxide-based composites are considered for diagnosis and treatment of cancer [9]. The nanocomposites can be used to kill the residual cancer cells which are left over after chemotherapy treatment and radiation therapy [10]. Several studies have reported the various biomedical applications of graphene oxide nanocomposites [11,12]. Lingyan et al. have designed aptamer-gold nanoparticle hybridized graphene oxide (Apt-AuNP-GO) which can be used for near-infrared (NIR) light-activatable photo thermal therapy [13]. Self-assembled Apt-AuNP-GO nanocomposites can be used to selectively target MUC1-positive (type I Trans membrane mucin glycoprotein) human breast cancer cells (MCF-7), due to the interaction of MUC1-binding-aptamers and the MUC1 on cell membranes. Additionally, this composite also exhibits high light-to-heat conversion capability for photo absorption of NIR light and it is effective at an ultra-low concentration, without damaging healthy cells [14]. The composite possesses specific targeting capability, excellent biocompatibility and tumor destruction which make it a potential agent for cancer treatment in NIR ranging photo thermal therapy [15]. Some reports also have illustrated that graphene oxide has the ability to kill cancer cells. This is remarkable as it opens new avenues for exploring the potential of graphene oxide for cancer therapy. Marco et al. have reported the effectiveness of graphene oxide alone as selective target of cancer stem cells [16]. They have employed a tumor sphere assay which measures the clonal expansion of single cancer stem cells under independent conditions. Through these assays, they have shown that graphene oxide can inhibit tumor-sphere formation in multiple cell lines like breast, ovarian, prostate, lung, and pancreatic. They have also shown that graphene oxide is ineffective in killing bulk cancer cells. Thus, graphene oxide can be used as an effective non-toxic therapeutic agent for killing cancer stem cells through differentiation based nanotherapy. GO-Fe_3_O_4_ nanocomposites were also tested as medical imaging and as therapeutic agents [3,17,18]. GO-Fe_3_O_4_ nanocomposites were mainly synthesized by chemical methods. These composites have the tendency to attain a reduced graphene oxide (RGO) nature, which causes changes in their chemical properties [9]. The study presented in this work employs the combination of chemical and ball milling methods for synthesis of the nanocomposite on a large scale. The ball mill method used in this study has the advantage of synthesizing the GO-Fe_3_O_4_ nanocomposite without changing the chemical structure of the graphene oxide frame work. Magnetite nanoparticles (Fe_3_O_4_) can be synthesized by various methods like coprecipitation, sol-gel, organometallic, and ball milling methods [19,20]. The GO (Graphene Oxide) framework will help in the formation of magnetic nanoparticle aggregates which will modify the magnetic behavior locally and which affects the hyperthermia efficiency [21]. Chen et al. have reported the synthesis of magnetite nanoparticles by ball milling of Fe_2_O_3_ in a wet medium [22]. They have shown the significance of water as a medium for milling in the formation of Fe_3_O_4_ phase as the dry milling of hematite does not form any magnetite phase. Using a ball mill method, nanocomposites can be synthesized on a large scale [23]. The magnetic property of the nanocomposite can be tuned by adjusting the milling time and by changing the composition used for the ball mill [24]. These nanocomposites can effectively be used as MRI contrast agents and in magnetic hyperthermia for the killing of cancer cells [25].

In this study we report a simple, efficient and scalable mechanochemical method for the synthesis of GO-Fe_3_O_4_ nanocomposite. The GO-Fe_3_O_4_ nanocomposite was synthesized by ball milling of Fe powder and graphene oxide in toluene medium. Interestingly the amount of graphene oxide used for the milling has a role in determining the formation of magnetic iron oxide phase. High amounts of graphene oxide resulted in the formation of hematite and magnetite. The magnetic properties required to obtain high SAR values (Specific Absorption Rate-Power absorbed per unit mass of the magnetic nanocomposite in the presence of applied magnetic field) for the composite were optimized by varying the milling time. Nanocomposites milled for different lengths of time have shown differences in magneto thermic heating behavior. The magnetic composites synthesized by the ball milled method were able to retain the functional groups of graphene oxide. The magnetic nanocomposites have been studied for the killing of HeLa cells.

## 2. Results and Discussion

### 2.1. Characterization of GO-Fe_3_O_4_ Nano-Composite

**Figure 1:**
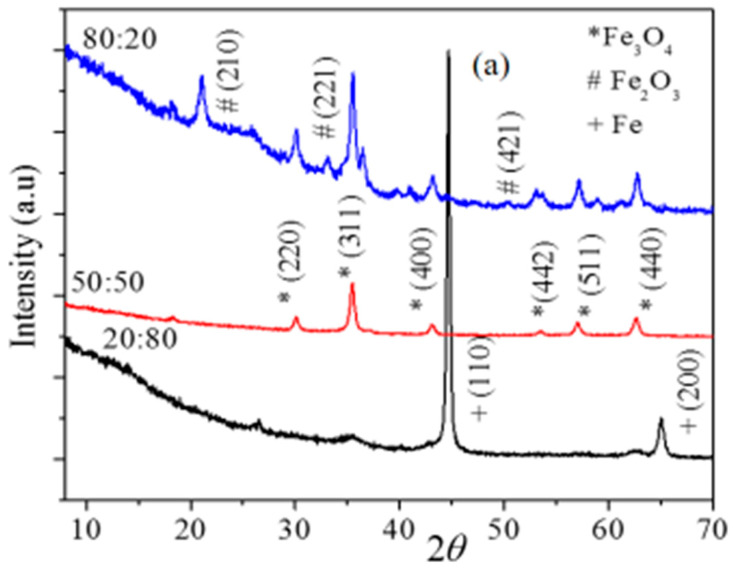

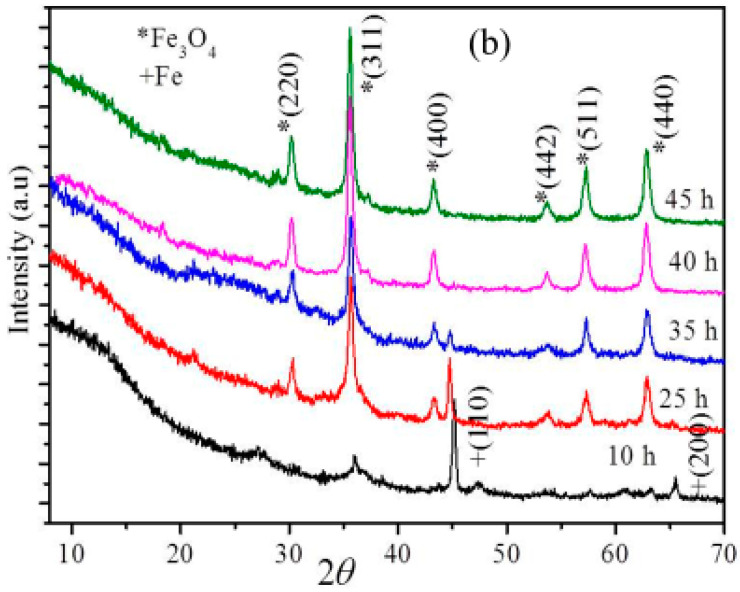
(**a**) X-ray diffraction (XRD) profiles obtained from milled graphene oxide and Fe nanocomposite with GO-Fe compositions 20:80, 50:50, and 80:20. (**b**) XRD patterns of GO-Fe (50:50) nanocomposite milled for 10, 25, 35, 40, and 45 h.

The nanocomposites obtained by the milling of the three different compositions of GO-Fe (20:80, 50:50 and 80:20) were washed with water by centrifugation at 5000 rpm and dried in an oven at 50 °C. XRD profiles obtained from the three different compositions of GO-Fe nanocomposites, which were milled for 45 h, are shown in Figure 1a. The XRD profiles in Figure 1a reveal diffraction peaks corresponding to Fe, Fe_3_O_4_ and Fe_2_O_3_, depending on the composition of GO and Fe used for milling. The magnetic phases obtained upon ball milling of metallic Fe powder and GO at similar conditions have resulted in different phases of iron oxide. The XRD pattern of GO-Fe with a 20:80 composition showed peaks corresponding to Fe and Fe_3_O_4_ phases. The peaks corresponding to Fe_3_O_4_ phase had less intensity compared to the Fe phase, which indicates that the presence of a lesser amount of graphene oxide results in suppressed oxidation of Fe, whereas in the case of a 50:50 composition, the Fe powder was completely oxidized into Fe_3_O_4_ phase [26,27]. In the case of the 80:20 composition, the nanocomposite had XRD peaks corresponding to both Fe_3_O_4_ and Fe_2_O_3_, which can be attributed to the role of graphene oxide. The formation of different magnetic phases was clearly related to the amount of graphene oxide used. The oxidation of the Fe powder can be attributed to the adsorbed oxygen and water trapped between the layers of graphene oxide. The oxidation extent of Fe is completely determined by the amount of graphene oxide. The 50:50 composition resulted in a pure Fe_3_O_4_ phase and thus it was used for the hyperthermia experiments in this work. The XRD patterns of these samples with several milling durations are shown in Figure 1b. From the XRD patterns it was evident that the Fe_3_O_4_ phase started forming at a milling time of 10 h, and appeared in all other samples at 25, 35 40 and 45 h of milling time. The average crystalline sizes for all the samples were calculated using FWHM (Full Width Half Maximum) of the highest intensity peak (311). The average crystalline sizes of the Fe_3_O_4_ nanoparticles was around 20 nm. At a 25 h milling time, a higher percentage of Fe_3_O_4_ phase than the Fe phase could be inferred by the intensity ration of highest intensity peaks (311) and (110) which correspond to Fe_3_O_4_ and Fe, respectively [28]. Upon continuation of milling, the intensity of XRD peaks corresponding to Fe decreased and eventually all the Fe was oxidized to Fe_3_O_4_ after 40 h of milling.

The following reactions are expected to take place during milling of Fe powder and graphene oxide in the presence of water in toluene medium, again these reactions are subjected to the properties of graphene oxide.

2 Fe + 3 H_2_O → Fe_2_O_3_ + 3 H_2_

3 Fe + 4 H_2_O → Fe_3_O_4_ + 4 H_2_

From the above reaction, it can be inferred that there is a formation of hydrogen in the vials from the reduction of water by iron powder.

The amount of hydrogen released from the reduction of water can be 600 cm^3^ per gram of Fe according to the stoichiometric reaction of Fe and water [29]. The hydrogen released during the process will be adsorbed on the graphene oxide or trapped in between the interlayers of graphene oxide which manipulates the formation of different magnetic phases.

### 2.2. Raman Analysis

**Figure 2:**
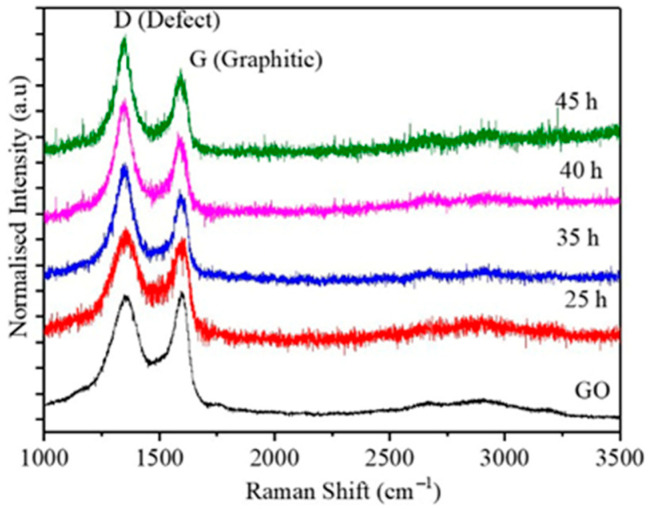
Raman spectra obtained from pure GO and GO-Fe nanocomposite with composition 50:50 milled for 25, 35, 40, and 45 h.

The GO-Fe nanocomposites with a 50:50 composition, which were milled for different times, were further characterized by Raman spectroscopy, the data is shown in Figure 2. The graphene oxide synthesized by Hummers method showed the D (Defect—structural defects created by the oxidation of Graphene frame work) and G (Graphitic—first order scattering of E_2g_) bands at frequencies ~1350 and 1588 cm^−1^, respectively. The ratio of the peak intensities corresponding to the D and G bands for the as prepared graphene oxide was 0.96. The peak positions and intensities ratio obtained for D and G bands agreed with the reported values of graphene oxide synthesized by Hummer’s method [30]. Upon milling, the peak positions of the graphene oxide corresponding to GO-Fe_3_O_4_ nanocomposite were approximately the same as that of the pure graphene oxide.

From the Raman data we obtained the intensity ratios of the D and G peaks which are listed in Table 1. The intensity of the D peak increased with milling time, which can be attributed to the increased defective structure upon milling. Since these samples were milled in toluene medium graphene oxide is subjected to further exfoliation which results in the formation of GO sheet with a smaller number of layers which will be further confirmed by AFM.

### 2.3. AFM Analysis of GO-Fe_3_O_4_ Nanocomposites

**Figure 3:**
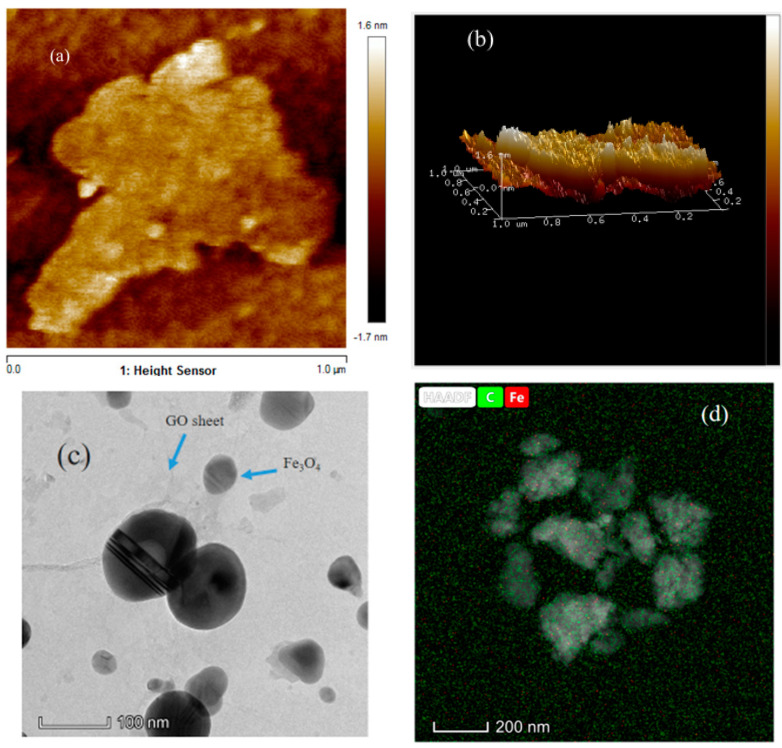
(**a**, **b**) Alternating magnetic field (AFM) images of 40 h milled GO-Fe with composition 50:50. (**c**) Bright field TEM images of 40 h milled sample. (**d**) Representative compositional mapping result obtained from the region of interest.

The nanocomposites obtained by ball milling were dispersed in water with sonication and drop dried on a glass slide under an IR lamp. The AFM images were recorded in tapping mode. The images of the 25 and 40 h milled samples are shown in Figure 3a,b. From the height profiles, it was clear that the thickness of the composite decreased with milling time, which again reaffirmed the observations made from the Raman data. This again shows that the graphene oxide exfoliation was induced by milling in the presence of toluene. The bright field TEM image is provided in Figure 3c, nanoparticles are arranged randomly on the GO sheet. STEM-high angle annular dark field (STEM-HAADF) image of the region of interest (nanoparticles over a large graphene sheet) carbon map and Fe map are provided in Figure 3d qualitatively shows that the Fe_3_O_4_ nanoparticles formed over the graphene oxide sheet.

### 2.4. Magnetic Measurements

**Figure 4:**
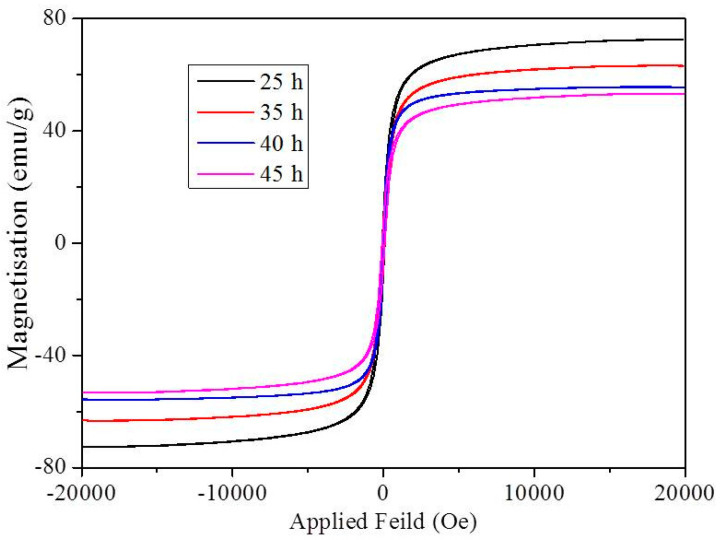
Magnetic hysteresis curves obtained for GO-Fe nanocomposite with a 50:50 composition milled for 25, 35, 40, and 45 h.

The magnetization (M) as a function of applied field (H) (M-H loops) was measured for the GO-Fe (50:50) with milling times of 25, 35,40 and 45 h at room temperatures, and are shown in Figure 4. M-H curves show that all the samples were superparamagnetic in nature. The saturation magnetization for the 25 h milled sample was the highest with a value of 78 emu/g. Saturation magnetization of the nanocomposites decreased with an increase in milling time from 25–40 h, which can be attributed to the decrease in Fe in the resulting composition. The decrease in the magnetization from milling times of 40 and 45 h was due to the size effect which can be attributed to the magnetically disordered surface spins, higher milling times makes the magnetic nanoparticles finer.

### 2.5. Magneto-Thermal Ability of GO-Fe_3_O_4_ Nanocomposite for Killing of HeLa Cells

**Figure 5:**
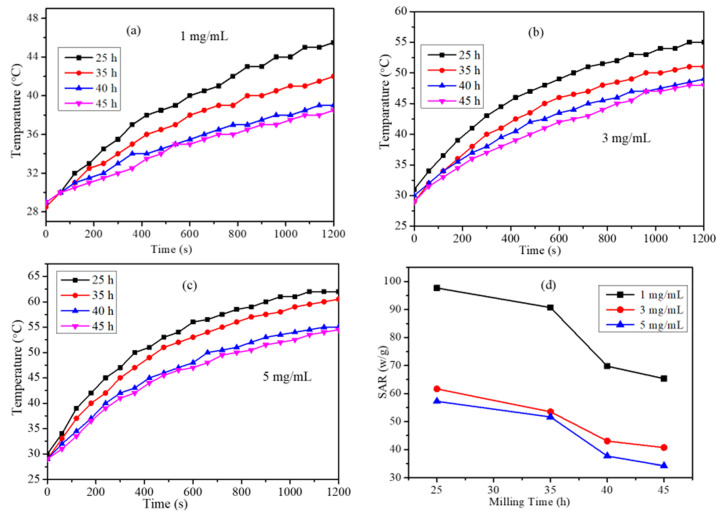
(**a**–**c**) Temperature vs. time for GO-Fe nanocomposite with a 50:50 (GO: Fe) composition milled for 25, 35, 40, and 45 h, and (**d**) Specific Absorption Rate (SAR) vs. milling time.

The GO-Fe (50:50) nanocomposites synthesized from the ball mill method were examined for their magneto thermal ability to raise the temperature of water. The increase in the temperature of water after applying an alternating magnetic field (AMF) with magnitude 3.6 kA/m and frequency 236 kHz was recorded using an alcohol thermometer. The magneto thermic data of the samples milled to different times (25–45 h) was obtained for 1, 3 and 5 mg/mL concentrations (Figure 5). SAR values were calculated for all the samples using equation (1). The SAR vs. milling time plot is shown in Figure 5d. The trend in the variation of SAR values with respect to milling time showed a composition dependent SAR. The 25 h milled composite showed the highest SAR value for all concentrations it decreases with further milling. This shows that the magnetic heating caused by the nanocomposite depends on the milling time. Since the 40 h milled sample had a pure Fe_3_O_4_ magnetic phase and better SAR value compared to the 45 h milled sample, the in vitro studies for the killing of cancer cells were performed using this sample.

Although 25 h milled sample had the highest SAR values at all concentrations, it was not considered for the biological studies because Fe might undergo further oxidation. Hence a stable magnetite graphene oxide phase GO-Fe (50:50) at 40 h was chosen for further studies.

### 2.6. Biocompatibility of GO-Fe_3_O_4_ Magnetic Nanocomposite

**Figure 6:**
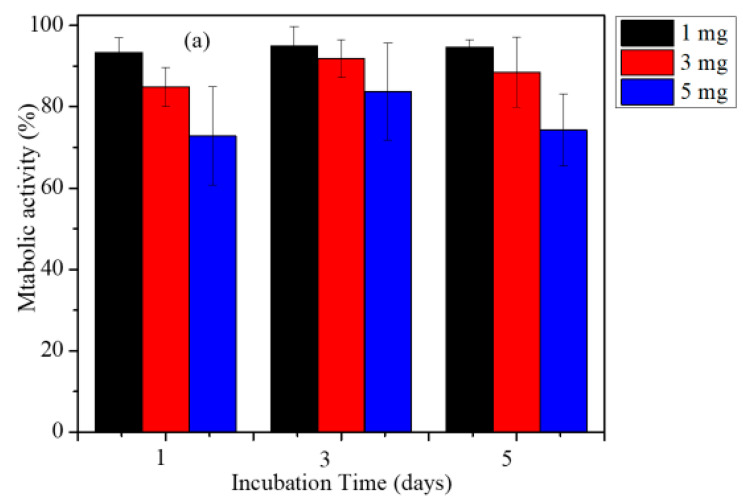

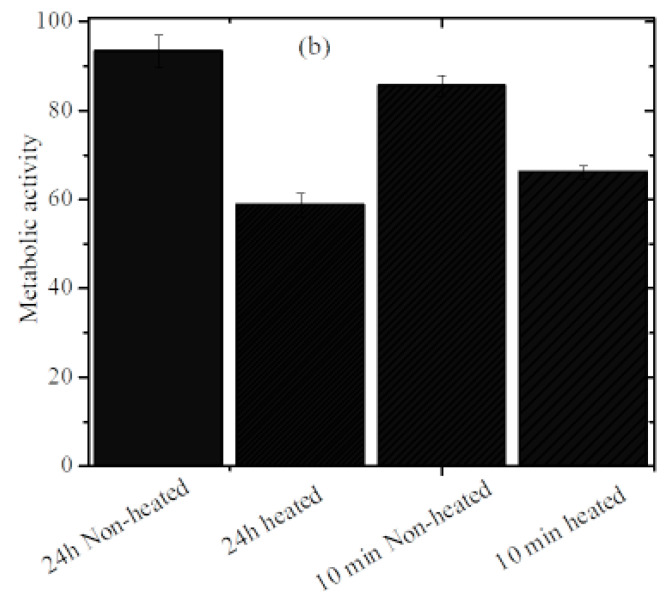
(**a**) MTT assay of HeLa and (**b**) HeLa cells treated with GO-Fe_3_O_4_ nanocomposite and subjected to an AC magnetic field.

Biocompatibility of the nanocomposites was determined by using an MTT assay. The MTT data obtained for a 40 h milled sample with HeLa cells are shown in Figure 6.

The biocompatibility of the water dispersible graphene oxide nanocomposites was obtained by quantifying the metabolic activity of the cells using MTT assay. The MTT assay shows that the nanocomposites are biocompatible, as 90 % cells survived upon exposure to the nanoparticles for 24 h. To determine the magneto thermal ability to kill cancer cells, 1 mg of GO-nanocomposite was incubated with the HeLa cells for 24 h. The cells were subjected to Alternating Magnetic Field (AMF) using an Ambrell Easy heat induction heater for 10 min. Immediately after AC magnetic field treatment, MTT solution (0.5 mL) was added to each well and incubated for 2 h. Metabolic activity was determined for magnetic field treated (heated) and non-treated HeLa cells. The magnetic nanocomposite killed around 40% of the HeLa cells with a 1 mg/mL nanocomposite concentration. The structural changes in Hela cell morphology upon treatment with magnetic field was studied by fluorescence imaging.

### 2.7. Morphological Studies of HeLa Cells

**Figure 7:**
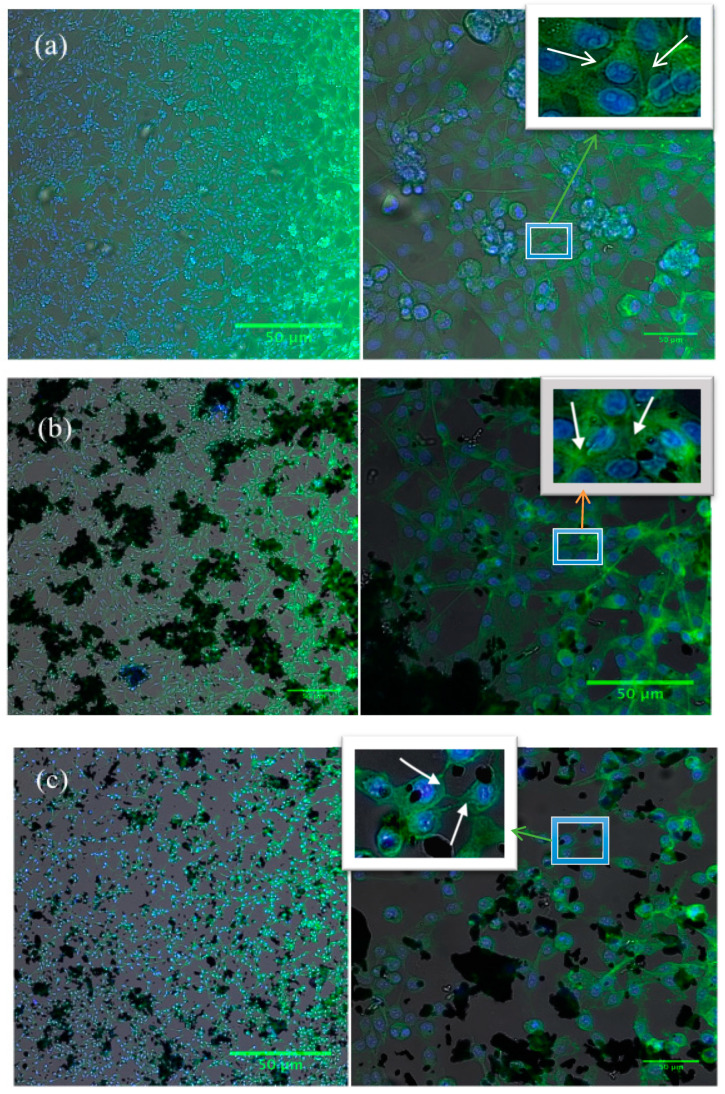
(**a**) Fluorescence images of Control, (**b**) Fluorescence images of HeLa cells treated with GO-Fe_3_O_4_ non-heated (not subjected to AC magnetic field) and (**c**) Fluorescence images of HeLa cells treated with GO-Fe_3_O_4_ heated (subjected to AC magnetic field). The blue box shows the portion of the image selected for magnification while the white box shows magnified view of the selected portion. The green (**a**), (**c**) and orange (**b**) arrows point to the magnified view while the white arrows point to the cytoskeleton changes.

The morphological changes in the shape of the HeLa cells upon the treatment with GO-Fe_3_O_4_ nanocomposite were studied using florescence microscopy. From the images (Figure 7) it was visible that upon treatment with graphene oxide nanocomposite, the cell morphology changed. There was a visible change in actin filaments and in the structure of the cell nucleus. The composite has shown slight variation in its response to the cells upon application of external magnetic field. The images of the heated cells showed more cells with more damaged cytoskeletons, and cells had attained a spherical shape. Also, the graphene oxide sheet attained a membrane like structure upon exposure to the external magnetic field. The presence of nanoparticles on the sheet will enhance the effect of cell and GO interactions that will cause the destruction of the cytoskeleton that in turn will help in killing the cancer cells.

## 3. Materials and Methods

### 3.1. Synthesis of GO-Fe_3_O_4_ Nanocomposite

Graphene oxide was synthesized by Hummer’s method [31]. The Graphene oxide synthesized in the first step was dried in the oven at 50 °C, then 2.5 g of GO was mixed with 2.5 g of iron powder in the chromium steel vial of the planetary ball mill instrument (Eloquent Technologies, Bangalore, India). We added to the chromium steel vial six stainless steel balls, weighing a total of 100 g, to maintain the ball-to-powder ratio at 20:1. Toluene was used as the medium for milling, and the samples were extracted at 25, 35, 40, and 45 h. Three different compositions of GO-Fe by weight percent (20:80, 50:50 and 80:20) were used for milling. The samples extracted were washed using water by centrifugation and dried in an oven at 50 °C for 3 h.

### 3.2. Magneto Thermal Property of GO-Fe_3_O_4_ Nanocomposites

The magnetic nanocomposites synthesized by the ball mill method were dispersed in water by sonication and their heating ability was measured by Ambrel Easy Heat induction. The nanocomposite dispersion was placed at the center of the induction coil and an alternating current of 3.6 kA/m and a frequency of 236 kHz were applied for up to 30 min. The change in temperature was measured using an alcohol thermometer. The nanoparticle-induced increase in the temperature of the water dispersion enabled the calculation of SAR [32]:

(1) $$\mathrm{SAR}(W/g)=\frac{C}{m}\frac{\mathrm{dT}}{\mathrm{dt}}$$

dT/dt: initial slope of the temperature vs. time, where C is the specific heat capacity of water C_water_ = 4186 JL^−1^K^−1^, and m is the mass concentration of the magnetic material in g/L.

The hyperthermia effect of magnetic nanocomposites for the killing of HeLa cells were examined by dispersing 1 mg of magnetic nanocomposite in the cell medium, and the cells were exposed to an alternating magnetic field (AMF) for about 10 min. The metabolic activity of the cells was studied by MTT assay.

### 3.3. Cell Culture

HeLa cells (ATCC) were cultured in a 25 cm^2^ culture flasks in Dulbecco’s Modified Eagle Medium (DMEM) with 10% FBS (F2442 Sigma Aldrich, Saint Louis, MO, USA) and 1% antibiotic solution (antibiotic-antimycotic solution, Sigma Aldrich). Cells were trypsinized using 0.25% Trypsin-EDTA solution (T4049 Sigma Aldrich) and the number of cells were counted using haemocytometer (Improved Neubaner cell counter). The seeded cells were incubated at 37 °C in a CO_2_ incubator (New Brunswick^TM^ Galaxy 170S, Edison, NJ, USA) with 5% CO_2_ and 90% humidity.

### 3.4. Metabolic Activity Assessment (MTT Assay)

The biocompatibility of the magnetic nanocomposite was determined by quantifying the metabolic activity of the cells using MTT assay. The magnetic nanocomposites were sterilized by exposing them to UV light for 10 min. Tissue culture plate was seeded with 3 × 10^4^ cells per well and incubated for 24 h. After 24 h incubation, 1 mg/mL composite solution was added to the wells and the cells were subjected to Alternating Magnetic Field (AMF) using Ambrell Easy heat induction heater for 10 min. Immediately after treatment, MTT solution (0.5 mL) was added to each well and incubated for 2 h. The insoluble formazan crystals formed were dissolved in dimethyl sulphoxide (DMSO) and the optical density was recorded at 570 nm using a plate reader (Tecan Infinite M 1000 Pro., Durham, NC, USA).

### 3.5. Cell Imaging (Confocal Microscopy)

The cell morphology after GO-Fe_3_O_4_ nanocomposite treatment with and without AMF exposure was studied using confocal microscope (In Cell Analyzer 6000, GE healthcare life science, Issaquah, WA, USA). The images were acquired for both treated HeLa cells and normal HeLa cells, i.e, without AMF treatment. The composite treated cells were fixed with 3.7% Formaldehyde in 1× PBS for 20 min followed by cell permeabilization using Triton-X-100 solution in 1× PBS. Subsequently, nonspecific binding of proteins to the dye was inhibited by treating cells with 5% FBS in 1× PBS for 1 h, followed by staining of actin filaments and cell nuclei using ActinGreen 488 ready probes reagent and Hoechst 33258 dyes, respectively.

### 3.6. Flow Cytometry

The HeLa cells were incubated with magnetic nanocomposite for 24 h and exposed to AMF with NPs (Nanoparticles) for 20 min. The cells were stained using standard staining protocol and data was acquired by using BD FACS Verse^TM^ (Franklin Lakes, NJ, USA). The acquired data was analyzed using De novo FCS Express 6 software. Alexa Fluor 488 labelled annexin V and PI dye were used to distinguish apoptotic and healthy cells. PI is a fluorescent dye that gets intercalated between the base pairs of double stranded DNA of dead cells but excluded by cell membrane of live cells under normal conditions. Annexin V binds easily to the surface exposed phospatidylerine (PS) due to phospholipid asymmetry in case of apoptotic cells. 1 × 10^5^ cells were seeded per well and incubated for 24 h with nanocomposite. After 24 h, the cells were harvested and suspended in 1 × Annexin binding buffer. Then 5 μL Annexin V and 1 μL PI dye was added and incubated for 15 min. Afterwards 400 μL of Annexin binding buffer was added and the vials were kept in ice until analysis. The unstained and negative controls were also maintained for analysis. A blue laser with excitation wavelength 488 nm was used for Annexin V and PI having emission wavelengths in the range of 511–543 nm, and 565–617 nm, respectively.

### 3.7. Characterization of the GO-Fe_3_O_4_ Composite

X-ray diffraction (XRD) profiles from as-synthesized samples were obtained by using an X-Pert PAN Analytical machine employing a Cuk_α_ radiation source. Magnetic measurement data from the as-synthesized composites were conducted by using a Lakeshore vibrating sample magnetometer (VSM). Raman spectra from as-synthesized samples were obtained using a microscope setup (HORIBA JOBIN YVON, Lab RAM HR, Minami-ku Kyoto, Japan) consisting of a diode-pumped solid-state laser operating at 532 nm with a charge coupled detector. A 300 kV FEI TITAN transmission electron microscope (TEM) was used for imaging the samples, for obtaining STEM-EDS and STEM-high angle annular dark field (STEM-HAADF) images and was used for compositional analysis of the samples. The ball milling of the Fe powder and graphene was done using the chromium steel vial of the planetary ball mill instrument (Eloquent Technologies, Bangalore, India).

## 4. Conclusions

In this study, we synthesized a GO-Fe_3_O_4_ nanocomposite on a large scale by mechanochemical ball milling of graphene oxide and iron powder. The nanocomposite synthesized were characterized by XRD, Raman spectroscopy, AFM, and magnetic property analysis. The magnetic phases formed upon mechanochemical milling depended on the initial compositions of GO and Fe powder used and milling time. The nanocomposites were further studied for hyperthermia applications for the killing of HeLa cancers cells under AMF. SAR values of nanocomposites were determined using 1, 3, and 5 mg/mL concentrations. The SAR showed a composition dependency on the magnetic phases. Of the nanocomposites, one was studied for the killing of HeLa cancers cells with an applied AC magnetic field. The nanocomposite was able to kill around 40% of the cells with an exposure time of 10 min. The change in cytoplasm morphology of the HeLa cells were further studied by fluorescence imaging.

## Figures and Tables

**Table 1:** Intensity ratio of defect and graphitic peaks of graphene oxide.

Sample Composition	I_D_/I_G_
GO	0.96
25 h	1.10
35 h	1.29
40 h	1.37
45 h	1.40

**I_D_/I_G_** = D intensity/G intensity ratio
