# Correction: Narayanaswamy et al. Synthesis of Graphene Oxide-Fe_3_O_4_ Based Nanocomposites Using the Mechanochemical Method and In Vitro Magnetic Hyperthermia. *Int. J. Mol. Sci.* 2019, *20*, 3368

**DOI:** 10.3390/ijms26157278

**Published:** 2025-07-28

**Authors:** Venkatesha Narayanaswamy, Ihab M. Obaidat, Aleksandr S. Kamzin, Sachin Latiyan, Shilpee Jain, Hemant Kumar, Chandan Srivastava, Sulaiman Alaabed, Bashar Issa

**Affiliations:** 1 Department of Physics, United Arab Emirates University, Al-Ain 15551, United Arab Emirates; venkateshnrn@gmail.com; 2 Ioffe Physical Technical Institute, St. Petersburg 194021, Russia; askam@mail.ioffe.ru; 3 Center for Biosystems Science and Engineering, Indian Institute of Science, Bangalore 560012, India; sachinlatiyans@gmail.com (S.L.); shilpeejain@iisc.ac.in (S.J.); 4 Materials Engineering, Indian Institute of Science, Bangalore 560012, India; hemant.msme95@gmail.com (H.K.); csrivastava@iisc.ac.in (C.S.); 5 Department of Geology, United Arab Emirates University, Al-Ain 15551, United Arab Emirates; s.alaabed@uaeu.ac.ae; 6 Department of Medical Diagnostic Imaging, College of Health Sciences, University of Sharjah, Sharjah P.O. Box 27272, United Arab Emirates; bissa@sharjah.ac.ae

In the original publication [1], minor mistakes were present in Figure 2 and Table 1. The plots for both the 35 and 40 h milling times are identical. This error occurred during data transfer from the text file to the original software. Instead of copying the data from the 35 h text file (to plot the Raman spectroscopy for the 35 h milling time), the data was copied from the 40 h text file. Hence, the data for the 40 h milling time was copied twice, and this resulted in identical curves for the results at 35 h and 40 h. We did not notice this because the difference in the data for each sample is minimal. Using the original data for the 35 h milling time, we replotted the curve and corrected Figure 2. 

The corrected Figure 2 and Table 1 appear below. The authors state that the scientific conclusions are unaffected. This correction was approved by the Academic Editor. The original publication has also been updated.

## Figures and Tables

**Figure 2:**
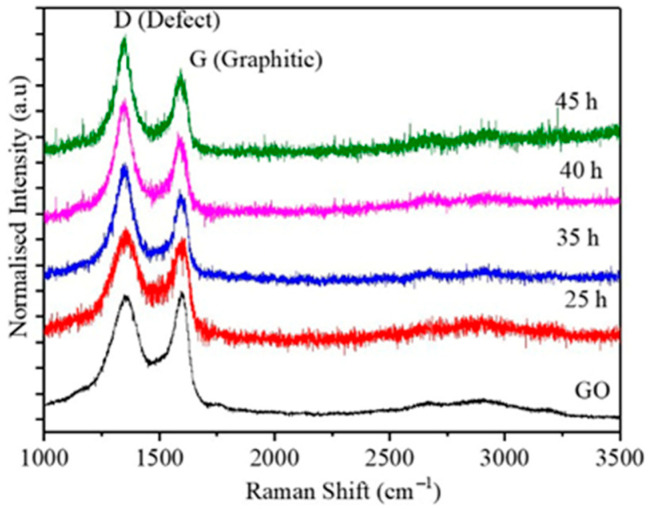
Raman spectra obtained from pure GO and GO-Fe nanocomposite with composition 50:50 milled for 25, 35, 40, and 45 h.

**Table 1:** Intensity ratio of defect and graphitic peaks of graphene oxide.

Sample Composition	I_D_/I_G_
GO	0.96
25 h	1.10
35 h	1.29
40 h	1.37
45 h	1.40

**I_D_/I_G_** = D intensity/G intensity ratio
